# Type A *Francisella tularensis* Acid Phosphatases Contribute to Pathogenesis

**DOI:** 10.1371/journal.pone.0056834

**Published:** 2013-02-15

**Authors:** Nrusingh P. Mohapatra, Shilpa Soni, Murugesan V. S. Rajaram, Kristi L. Strandberg, John S. Gunn

**Affiliations:** Department of Microbial Infection and Immunity, Center for Microbial Interface Biology, The Ohio State University, Columbus, Ohio, United States of America; Université Paris Descartes; INSERM, U1002, France

## Abstract

Different *Francisella* spp. produce five or six predicted acid phosphatases (AcpA, AcpB, AcpC, AcpD, HapA and HapB). The genes encoding the histidine acid phosphatases (*hapA*, *hapB*) and *acpD* of *F. tularensis* subsp. Schu S4 strain are truncated or disrupted. However, deletion of HapA (FTT1064) in *F. tularensis* Schu S4 resulted in a 33% reduction in acid phosphatase activity and loss of the four functional acid phosphatases in *F. tularensis* Schu S4 (ΔABCH) resulted in a>99% reduction in acid phosphatase activity compared to the wild type strain. All single, double and triple mutants tested, demonstrated a moderate decrease in mouse virulence and survival and growth within human and murine phagocytes, whereas the ΔABCH mutant showed >3.5-fold decrease in intramacrophage survival and 100% attenuation of virulence in mouse. While the Schu S4 ΔABCH strain was attenuated in the mouse model, it showed only limited protection against wild type challenge. *F. tularensis* Schu S4 failed to stimulate reactive oxygen species production in phagocytes, whereas infection by the ΔABCH strain stimulated 5- and 56-fold increase in reactive oxygen species production in neutrophils and human monocyte-derived macrophages, respectively. The ΔABCH mutant but not the wild type strain strongly co-localized with p47*^phox^* and replicated in macrophages isolated from p47*^phox^* knockout mice. Thus, *F. tularensis* Schu S4 acid phosphatases, including the truncated HapA, play a major role in intramacrophage survival and virulence of this human pathogen.

## Introduction

Tularemia is a potentially fatal systemic disease of humans and animals caused by the bacterial pathogen *Francisella tularensis* subsp. *tularensis* (*F. tularensis*). The disease can be transmitted by ticks, biting flies, mosquitoes, water exposure, food, or aerosols and primarily occurs in the northern hemisphere including North America, Europe, and Asia. Small mammals (particularly rabbits) act as a reservoir for this bacterium *F. tularensis* is a facultative intracellular pathogen of macrophages and neutrophils, as well as nonphagocytic cells such as hepatocytes and airway epithelial cells [1]–[6]. Tularemia in humans is an acute febrile disease that shows cutaneous, oculoglandular, pneumonic, gastrointestinal or septic features depending upon the route of infection [7]–[10]. Only two of the four subspecies of *Francisella tularensis* (subsp. *tularensis, holarctica*) cause significant disease in immuno-competent humans. Infections with Type A *F. tularensis* are associated with the highest mortality rates. All *Francisella* subspecies exhibit greater than 95% DNA identity [11]. An attenuated live vaccine strain (LVS) of *F. holarctica* was isolated and has been used extensively in humans, but has not achieved approval for use in the United States [12].

Less than 10 CFU of a Type A strain is sufficient to cause the pneumonic form of tularemia in humans. This is characterized by rapid multiplication inside the cytosol of infected cells and subsequent damage to host tissues and organs, thereby disrupting their normal functions and inducing host cellular inflammatory responses [13]. *F. tularensis* enters the host macrophage by an asymmetric pseudo loop that is dependent upon serum complement and host cell receptors [14]–[18]. Once the bacterium is inside the host cell, it arrests phagosome maturation and the bacterial-containing vacuole transiently acidifies, which leads to the escape of the bacterium into the cytosol of the macrophage [19]–[21]. At later stages of cellular infection, the bacterium can be found in vesicles that are likely related to autophagy [22], [23]. Bacterial release from the host cells is thought to occur following *Francisella*-induced apoptosis [24]–[27] and pyroptosis [28], [29]. However, the complete intracellular life cycle of *F. tularensis* is not clearly understood.

Very few virulence factors have been identified in *Francisella* and the molecular events accounting for the development of tularemia are unclear. Several studies have indicated that the products of the *Francisella* pathogenicity island (FPI) genes either directly or indirectly contribute to the virulence of this pathogen [30]–[35]. Regulation of the FPI is controlled by several global regulators including MglA, SspA, PmrA, FevR, Hfq, RipA and MigR [36]–[47]. In addition to the FPI genes, *Francisella* acid phosphatases have been shown to play a key role in several virulence related properties [23], [48], [49]. The published genome sequence of *F. tularensis* Schu S4 possesses six acid phosphatases (*acpA, acpB, acpC* and truncated *acpD*, *hapA*, and *hapB)* [50]. Acid phosphatases are ubiquitous in nature, hydrolyze phosphomonoesters at acidic pH and have been associated with pathogen survival inside the phagosome through the inhibition of respiratory burst [51]–[58]. In our previous studies, we observed that the combined deletion of AcpA with AcpB, AcpC and HapA in *F. novicida* resulted an attenuated strain that was 100% protective against homologous challenge in the mouse model. Additionally this mutant did not escape from the macrophage phagosome, had negligible phosphatase activity, and failed to suppress the oxidative burst in human phagocytes [23], [49]. Transcriptional analysis demonstrated that expression of *acpA* and *hapA* increased at initial stage of the macrophage infection [23], and an *in vivo* proteomic analysis of *Francisella* infected mice spleens showed that there was 2-fold more AcpA protein isolated from these organs compared to the bacteria grown in broth [59]. Pierson et al. observed that *F. novicida* and *F. philomiragia* form outer membrane vesicles and these vesicles carry several hundred proteins, with AcpA one of the major proteins present [60]. A comparative proteomic profiling of culture filtrates of various *Francisella* spp. showed that all secreted AcpA *in vitro* [61]. Furthermore, a recent study performed by Dai et al. demonstrated that *Francisella* spp. AcpA is secreted into the culture supernatant *in vitro* and also secreted and translocated across the phagosomal membrane into the host cell cytosol at early stages of *F. novicida* and *F. tularensis* Schu S4 infection [62]. In this study, deletion of AcpA, AcpB, AcpC and truncated HapA in *F. tularensis* Schu S4 resulted loss of more than 99% of the acid phosphatase activity, more than a 3-log decrease in survival and growth inside human and murine macrophages and attenuation in the mouse model. Additionally we examined ROS induction by this mutant and demonstrated that it co-localized with NADPH oxidase components in human neutrophils and monocyte derived macrophages and lacked the wild type strain's ability to suppress the oxidative burst.

## Materials and Methods

### Growth conditions, strains and plasmid construction

All experiments were performed in Modified Mueller Hinton media (MMH): Mueller-Hinton medium supplemented with 0.1% glucose, 0.025% ferric pyrophosphate and 2% IsoVitaleX (Becton Dickinson, Cockeysville, MD) or on CHOC II plates incubated at 37°C and 5% CO_2_. Kanamycin was added at a concentration of at 15 mg/L when required. Primers, bacterial strains and plasmids are described in Table 1. The Type A virulent strain, *F. tularensis* subsp. *tularensis* Schu S4 was obtained from Rick Lyons (University of New Mexico, Albuquerque, USA). Schu S4 infections were conducted by CDC approved select agent users in facilities at The Ohio State University in accordance with local and national biosafety plans and procedures.

**Table 1:** List of primers, strains and plasmids.

	Primer sequences
JG1795	acgcgtcgacGGAGTTAGTGATTTAGTTGCAATAGGTGTT GC
JG1796	cgcggatccGCTTCATATGATACCTTTAGTTGTTAGATTCAAAGGAAATATTAATAAC
JG1797	cgcggatccAAATATTTACTCGGTAAGTTGCTTTAATCTAGTATTTTCGC
JG1798	tccccccgggGCTAAAGATAAGGGCATAAAGACTATCAAAGAGAG
JG1827	aaacgagctcgATGGGGCTGATTTGAGATCGGTAC
JG1828	tccccccgggAGAATTATTTTAGACCTAATTCCTTTAGTCTCTCGTTAAGA
JG1829	tccccccgggTATACTGATTTTGATTTAAAGATTAGAAAATTACCTAAAAATATTAAAATAGCA
JG1830	acgcgtcgacAATTGAGCTTGGAAAACGAATTAGTCCTTCAC
JG1831	tccccccgggAAGCTCGTGGAATACTTTTTCTTGAGCT
JG1832	cgcggatccTGATGTTTCTAAGTTCTTTTTCTTATGATGTTGATACCAT
JG1833	cgcggatccTAGAGGATTTTTGTAGTGGCGAAATTATATTTTAGATATTCG
JG1834	acgcgtcgacTAAGTATTTGTTTACTAATCACACCCTTCATAATAGCG
JG1839	tccccccgggTCGTAAATACTAGTAGCAATACAATTTCAAGAACTGAAAAG
JG1840	cgcggatccAATTGATTTTTGTTGTAATACCTATTATTTAATCCAATCGCG
JG1841	cgcggatccCCTCTCACTCTCAATACGATTAAATACATCTGC
JG1842	acgcgtcgacAAGAGATATTTAAACATTGCCAAGAGAAAGGTTATATTGAT
JG2007	cggaattccactgcagaggagggtttttaATGAAGCTCAATAAAATTACTTTAGGAATTTTAAGTC
JG2008	ccgctcgagGTTTAATTTATCCACTACTAATCCTGTCTTAGG
JG2009	ccgctcgagcactgcagaggagggtttttaATGAGAAAAATATTCACTATCGGCATTTTAAC
JG2010	cgcggatccTGTTGGTGTAGAAACTTTAGTGTCG
JG2169	CTAGCTAGCAGGAGACATGAACGATGAACATC
JG2170	GGGACGTCGGATTCACCTTTATGTTGATAAG
JG2171	ATCAGCTCACTCAAAGGCGG
JG2172	GGGACGTCGATTAAGCATTGGTAACTGTCAGACC

Strains	
Wild type *F. tularensis* Schu S4	From Dr. Rick Lyons
*acpA* deletion in Schu S4	This study
*acpB* deletion in Schu S4	This study
*acpC* deletion in Schu S4	This study
*hapA* deletion in Schu S4	This study
*acpA*, *acpB* deletion in Schu S4	This study
*acpA*, *acpC* deletion in Schu S4	This study
*acpB*, *acpC* deletion in Schu S4	This study
*acpA*, *hapA* deletion in Schu S4	This study
*acpC*, *hapA* deletion in Schu S4	This study
*acpB*, *hapA* deletion in Schu S4	This study
*acpA*, *acpB*, *acpC* deletion in Schu S4	This study
*acpA*, *acpC*, *hapA* deletion in Schu S4	This study
*acpB*, *acpC*, *hapA* deletion in Schu S4	This study
*acpA*, *acpB*, *acpC*, *hapA* deletion in Schu S4	This study

Plasmids		
JSG3383	pJC84	[63]
JSG3384	pJC84-AcpAup-AcpAdn	This study
JSG3385	pJC84-AcpBup-AcpBdn	This study
JSG3386	pJC84-AcpCup-AcpCdn	This study
JSG3387	pJC84-Hapup-Hapdn	This study
JSG2818	pFNLTP6	[78]
JSG3552	pFNLTP6-AcpA	This study
JSG3553	pFNLTP6-Hap	This study
JSG3554	pFNLTP6-AcpA-Hap	This study

The suicide vector pJC84 (generously provided by Jean Celli, Rocky Mountain Laboratories, NIAID, NIH, Hamilton, MT, USA) was used to generate in-frame deletions of acid phosphatase genes in the *F. tularensis* Schu S4 strain. Deletion constructs of *acpA* (FTT0221), *acpB* (FTT0156), *acpC* (FTT0620) and *hapA* (FTT1064) were constructed as described by Wehrley et al. [63]. In brief, the ∼1100 bp upstream and downstream products of the gene of interest were PCR amplified and cloned into the pJC84 plasmid using suitable restriction sites. The details of the primers are described in Table 1. All plasmid constructs were verified by sequencing and glycerol stocks were frozen at −80°C for further use. The deletion constructs were transformed into Schu S4 by electroporation as described by McRae et al. [64]. The kanamycin-resistant transformants were tested for integration of the allelic replacement plasmid using primer combinations JG2169/2170 and JG2171/2172. The positive clones were subjected to sucrose counter selection as described earlier [64]. Sucrose-resistant clones were patched on MMH-kanamycin plates to verify loss of the kanamycin-resistance marker, and colony PCR was performed to detect clones with allelic replacement within the correct chromosomal locus, using primers JG1795/JG1798 and primers JG2007/JG2008 for the *acpA* deletion, primers JG1827/JG1830 and primers JG2171/2172 for the *acpB* deletion, primers JG1831/JG1834 and primers JG2171/2172 for the *acpC* deletion, and primers JG1839/JG1842 and primers JG2009/JG2010 for the *hapA* deletion. The genetic complementations of *acpA* and/or *hapA* were constructed in pFNLTP6-GroEL plasmid as described by Wehrley et al. [63]. In Brief, *acpA* and *hapA* genes were amplified from Schu S4 genomic DNA using the primer sets JG2007/2008 and JG2009/2010, respectively. The *acpA* amplified PCR product was cloned into an EcoRI/XhoI digested pFNLTP6-GroEL plasmid (pFNLTP-6-AcpA) and/or the *hapA* PCR product was cloned into XhoI/BamHI digested pFNLTP6-GroEL plasmid (pFNLTP6-HapA/pFLNLTP6-AcpA-Hap). The cloned plasmids were confirmed by sequencing and transformed into different strains by electroporation as described above [63]

### Acid phosphatase assay

Acid phosphatase activity of Schu S4 and the deletion mutants were measured essentially as described previously [23]. The strains were grown overnight in 3 ml of MMH broth, cultures were normalized and cells were pelleted by centrifugation at 8000×g and 4°C for 15 min. The cell pellets were washed with phosphate buffer saline (PBS) and resuspended in 1 ml of PBS and transferred into the 2 ml lysing matrix (1 micron beads) tubes and vortexed for 5 cycles of 2 min each. Tubes were placed in ice between each cycle. Lysates were collected and centrifuged at 8000×g and 4°C for 15 min to remove any unbroken cell debris. The total protein concentration of the samples was measured by the BCA protein assay kit (Thermo Fisher Scientific Inc., Waltham, MA), the protein content was normalized before measuring acid phosphatase activity. The acid phosphatase activity was measured using the 6,8-difluoro-4-methylumbelliferyl phosphate (DiFMUP, Invitrogen) substrate. 50 µL of the 200 µM DiFMUP working solution was added into 50 µl of sample or control and the reaction was incubated at room temperature for 10 min. The mean fluorescence unit of the samples and the control was determined by an ELISA reader setting of excitation at ∼360 nm and emission detection at ∼460 nm. The relative fluorescence unit (RFU) was converted into percentages of the phosphatase activity measured for the wild type strain.

### Intramacrophage survival assay

Murine alveolar J774.1 macrophages, phorbol myristate acetate-induced (PMA, 10 ng/ml) THP-1 macrophages, primary murine bone marrow-derived macrophages (BMDMs), human monocyte derived macrophages (hMDMs) and neutrophils were used to study the intracellular survival of *F. tularensis* Schu S4 and the Schu S4 acid phosphatase mutants. Murine bone marrow cells were isolated from femurs of 6- to10-week-old BALB/c (Harlan Sprague), C57BL/6 and congenic p47*^phox^* knockout mice (provided by Dr. Chandan Sen, The Ohio State University). In brief, bone marrow-derived macrophages were cultured in Iscove's Modified Dulbecco's Media (IMDM) containing 10% heat-inactivated FBS, 20% L cell-conditioned medium, 100 U/ml penicillin, and 100 µg/ml streptomycin at 37°C in a humidified atmosphere containing 5% CO_2_. After 5 days of incubation, cells were collected and plated in 24-well plates in IMDM containing 10% heat-inactivated FBS [49]. Using The Ohio State University Institutional Review Board approved protocol, heparinized blood samples were collected from healthy human donors and processed further to isolate the hMDMs and neutrophils as described by Mohapatra et al. [49].

Regarding *in vitro* infections, 2×10^5^ J774.1 macrophages, PMA induced THP-1 macrophages or primary BMDMs were seeded in 24-well tissue culture plates. The macrophage monolayers were infected with *F. tularensis* wild type Schu S4 and acid phosphatase mutants grown over night in MMH media at an MOI ∼50∶1 as described previously [49]. At various time points, macrophages were lysed with 0.05% SDS and plated on CHOC II plates to enumerate the colony forming units (CFU).

For phagocyte infection studies in hMDMs and neutrophils, *Francisella* strains were opsonized with 50% autologous serum for 30 min at 37°C and subsequently washed three times with defined (HBSS) buffer to remove excess serum. Opsonized *Francisella* were resuspended in appropriate buffer and kept on ice. Human MDMs and PMNs were infected with opsonized bacteria at an MOI ∼50∶1 and the intracellular survival of the bacterial strains were determined as described by Mohapatra et al. [49].

### Mouse virulence studies

Pathogen free, 6–8 week old female BALB/c mice (n = 5/group) were purchased from Harlan Sprague. Mice were housed in sterile micro isolator cages in the BSL-3 facility at The Ohio State University. Mice were housed and used in strict accordance with guidelines established by The Ohio State University Institutional Animal Care and Use Committee (IACUC), and all efforts were made to minimize animal suffering. The mice were anesthetized intra-peritoneally with 200 µl tribromoethanol anesthesia (2.5 gm 2,2,2-tribromoethanol, 5 ml 2-methyl-2butanol, 200 ml water), infected with wild type *F. tularensis* Schu S4 or the mutant strains via the intranasal route (25 µl of bacteria in PBS in the nares of each mouse, 3×10^3^ CFU total) for survival and vaccine studies. Animals were monitored twice daily for signs of morbidity. Lungs and spleens were collected from the infected mice at different time points and homogenized in sterile PBS to determine the bacterial burden. Bacterial colony counts in each organ were determined after 48 h of incubation at 37°C from the homogenate organs on CHOC II plates.

### Transmission electron microscopy

PMA-induced THP-1 macrophages were infected with wild type Schu S4 or the quadruple mutant at an MOI of 50∶1 in four-well chamber slides. At 30 min or two hours post-infection, wells were washed three times with PBS, and fresh medium containing 50 µg/ml gentamicin was added for 30 min. After gentamicin treatment, cells were washed three times with PBS and either fixed or incubated further in the presence of medium containing 10 µg/ml gentamicin for an additional 22 hr. At the chosen time points, the wells were washed and fixed immediately with 2.5% warm glutaraldehyde followed by a cocktail of 2.5% glutaraldehyde and 1% osmium tetroxide in 0.1 M sodium cacodylate (pH 7.3) for 15 min. The cells were then stained with 0.25% uranyl acetate in 0.1 M sodium acetate buffer (pH 6.3) for 45 min, and viewed by transmission electron microscopy using an FEI Technai G2 Spirit microscope at 60 kV. The intra-phagosomal or cytosolic bacteria were identified as described by Mohapatra et al. [23].

### Respiratory burst assays

Production of reactive oxygen species by human neutrophils and macrophages after *Francisella* infection was detected using a luminescent substrate in an ELISA reader as described previously [49]. Human serum-opsonized zymosan particles (MOI of 10∶1) and PMA (200 nM) were used as positive controls for ROS production. The inhibition of ROS production by *Francisella* spp. was tested by incubating phagocytes with *F. tularensis* for 10 min at 37°C prior to adding opsonized zymosan.

### Confocal microscopy

The co-localization of *Francisella* with the NADPH oxidase components in human neutrophils and hMDMs were detected by confocal microscopy as described by Mohapatra et al. [49]. Neutrophils (10^6^/well) on serum-coated coverslips were infected with serum-opsonized *Francisella* spp. At different time intervals, coverslips were washed with HBSS and fixed with 3.5% paraformaldehyde for 30 min [49]. After fixation, cells were washed and permeabilized with chilled methanol for 15 sec followed by HBSS washing and blocking with 20% normal human serum (Cambrex, Charles City, IA) and 5% BSA (blocking solution) for 2 hrs. Infected cells were treated with a primary antibody: mouse monoclonal anti-*F. tularensis* (1∶5000 dilution; BEI Resource) and/or rabbit anti-p47*^phox^* Ab or rabbit anti-gp91*^phox^* Ab (1∶5000 dilution; Santacruz Biotech, CA) for 2 hrs in blocking solution. Coverslips were washed and incubated with secondary Ab (goat anti-mouse Alexa Fluor 488 or donkey anti-rabbit Alexa Fluor 546, 1∶5000 dilution [Invitrogen]) for 1 hr. Coverslips were washed and mounted with Prolong anti-fade reagent (Invitrogen) and viewed with Olympus FV1000 spectral confocal microscope. Resting neutrophils or cells activated with 200 nM PMA for 5 min were used as negative and positive controls, respectively. Similarly, human MDMs were used to determine the co-localization of the bacterium with p47*^phox^*/gp91*^phox^* by confocal microscopy as described previously [49]. Quantification of co-localization of the bacteria with the NADPH oxidase components was determined as described by Mohapatra et al. [49].

The phosphorylation of p40*^phox^* and p47*^phox^* in cell lysates of human neutrophils and MDMs was detected as described previously by Mohapatra et al. [49]. In brief, neutrophils or hMDMs were infected with serum-opsonized Schu S4 strains at an MOI ∼50∶1 and the infection was synchronized by a brief incubation at 12°C. At different time intervals, uninfected and infected cells were lysed in macrophage lysis buffer. The cell lysates were boiled in Laemmli sample buffer, and equal amounts of proteins were separated by SDS-PAGE, transferred to a nitrocellulose membrane, and incubated with primary Ab against phospho-p40*^phox^* (Cell Signaling Technology, Beverly, MA; 1∶500 dilution) or phospho-p47*^phox^* (1∶1000 dilution) (Dang, 2006). This was followed by a goat anti-rabbit HRP-conjugated secondary Ab (Bio-Rad; 1∶1000 dilution) and development by ECL (Amersham/GE Healthcare Bio-Sciences, Piscataway, NJ). The ECL signal was quantified using a scanner and densitometry (Scion Image, Frederick, MD), as previously described [65].

## Results

### Construction of *F. tularensis* Schu S4 acid phosphatase gene deletions

The comparative genome analysis of *F. novicida* and *F. tularensis* subsp. *tularensis* revealed that AcpA (FTT0221), AcpB (FTT0156) and AcpC (FTT0620) were highly conserved (98.05, 98.97, and 96.76% identity, respectively). The genes encoding HapA, HapB, and AcpD are truncated/interrupted in *F. tularensis* subsp. *tularensis* Schu S4. This truncation has resulted in a lack of interest in HapA in Schu S4, driven by the fact that the predicted active site is in the C-terminal region that would be absent in the Schu S4 truncated protein. *acpD* and *hapB* are severely disrupted in Schu S4 [50] and thus were not further considered. In-frame deletion of genes creating single (*ΔacpA, ΔacpB, ΔacpC, ΔhapA*), double [*ΔacpA ΔacpC* (ΔAC), *ΔacpB ΔacpC* (ΔBC), *ΔacpA, ΔacpB* (ΔAB), *ΔacpA ΔhapA* (ΔAH), Δ*acpB* Δ*hapA* (ΔBH), Δ*acpC* Δ*hapA* (ΔCH)], triple [*ΔacpA ΔacpB ΔacpC* (ΔABC), *ΔacpA, ΔacpC ΔhapA* (ΔACH), *ΔacpB, ΔacpC ΔhapA* (ΔBCH)] and quadruple [Δ*acpA* Δ*acpB* Δ*acpC* Δ*hapA (ΔABCH)*] mutants in Schu S4 were generated using the SacB-assisted allelic replacement suicide vector pJC84, as described by Wherly et al. [63]. The lack of polar effects of the downstream genes of AcpC mutant was determined by RT-PCR (data not shown). Polar effects were not a concern for the other mutants based on their genomic arrangement. None of the constructed mutants demonstrated a growth defect in MMH broth or abnormal colony size/appearance on plates (data not shown).

### Acid phosphatase activity assays of mutant and wild type strains

**Figure 1:**
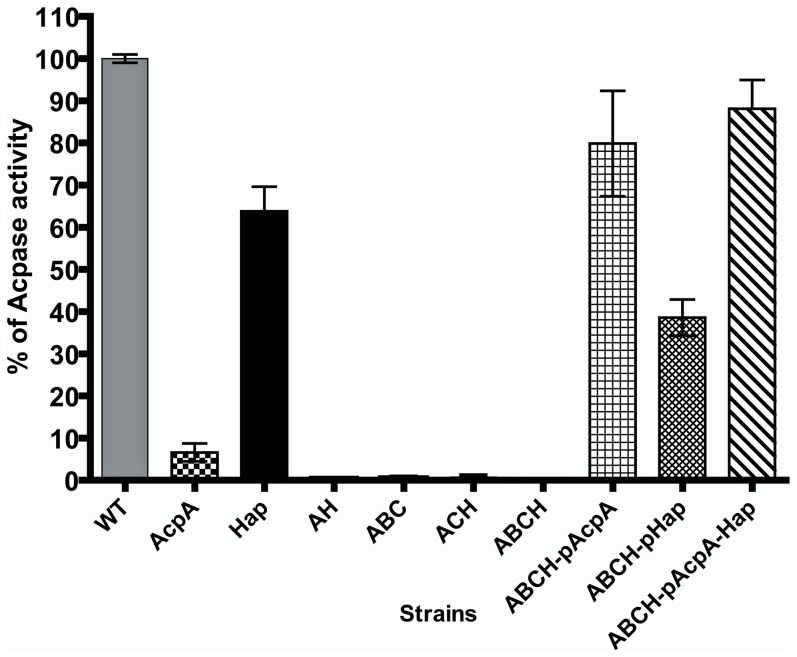
Acid phosphatase assay. Acid phosphatase activity was determined from whole cell lysates of *F. tularensis* acid phosphatase mutants by a fluorometric method using DiF-MUP as the substrate. The data are presented as percent of wild type phosphatase activity.

To examine their individual and additive contributions to Schu S4 acid phosphatase activity, strains lacking AcpA, AcpB, AcpC and HapA were examined. The enzyme activities were determined from the whole cell lysates by measuring the RFU, using difluoromethyl umbeliferyl phosphate (DifMUP) as the substrate. The RFU were displayed as a percentage of enzyme activity compared to the wild type strain (Fig. 1). Deletion of *acpA* or *hapA* decreased the total acid phosphatase activity by 89% and 33% respectively compared to the wild type strain. The reduction in acid phosphatase activity by deletion of *hapA* was surprising as this gene was predicted to produce a non-functional protein. The loss of *acpB* or *acpC* contributed 11% and 43% of the acid phosphatase activity respectively (data not shown). In triple deletion strains, we found that 93% and 95% of acid phosphatase activity was decreased in ΔABC and ΔACH mutants, respectively, compared to wild type strain. Acid phosphatase activity was completely abrogated in a ΔABCH mutant (>99.99%). The acid phosphatase activity of the ΔABCH mutant complemented with *acpA* or *hapA* or both genes recovered to 79, 38 and 88% of wild type enzyme activity, respectively (Fig. 1).

### Intramacrophage survival of acid phosphatase mutants

**Figure 2:**
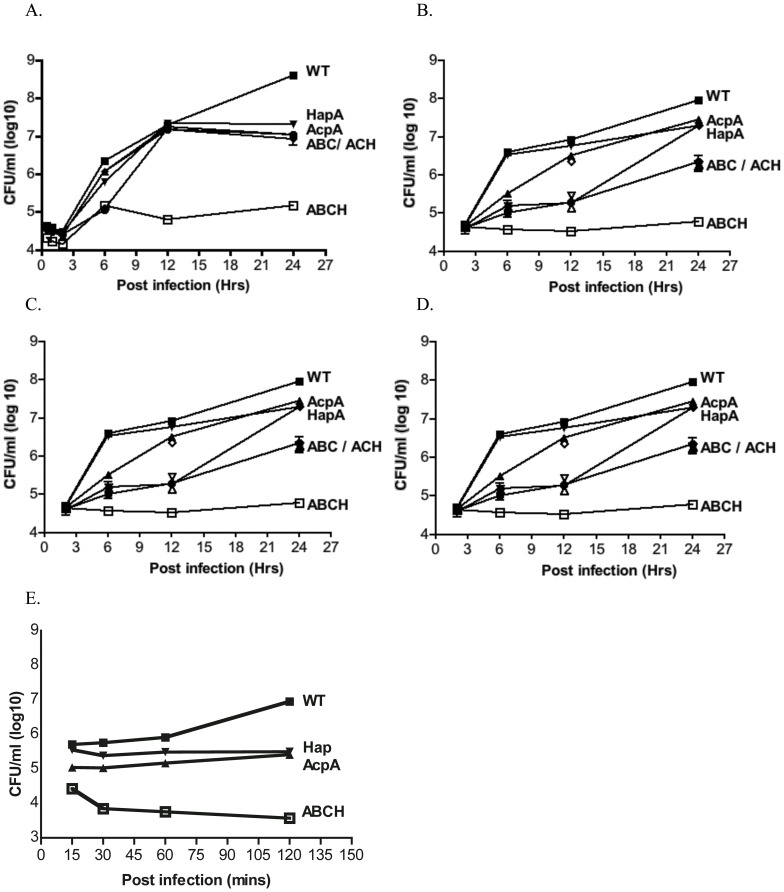
Intramacrophage survival of *F. tularensis* Schu S4 wild type and the acid phosphatase mutants in human and murine macrophages. (A) hMDMs, (B) PMA induced THP-1 macrophages, (C) bone marrow derived macrophages from wild type BALB/c mice, (D) J774.1 murine macrophages, or (E) human neutrophils were infected with *F. tularensis* Schu S4 (▪) or acid phosphatase mutants Δ*acpA* (▴), Δ*hapA* (▾), ΔABC (♦), ΔACH (•), or ΔABCH (□) strains.

Macrophages are the primary targets of the *F. tularensis* infection in mammals, and our previous study demonstrated that *F. novicida* acid phosphatases contribute to survival in human and murine macrophages [23], [48]. To determine if the Schu S4 acid phosphatase mutants similarly contribute to growth and survival in human and murine macrophages, we infected these phagocytes with various acid phosphatase mutants and the bacterial CFU were determined from 15 min to 24 hrs post-infection. The *ΔacpA* and *ΔhapA* mutant showed >1 log reduction in CFU compared to the wild type macrophages at 24 hrs post infection in hMDMs, the human macrophage-like cell line THP-1, BMDMs (from BALB/c), and J774.1 mouse macrophage cell line (Fig. 2A–D). The ΔABCH mutant exhibited a 3–4 log decrease in the number of CFU at 24 hrs post infection in all macrophages examined, while ΔABC and ΔACH mutants showed a >2 log decrease in the number of CFU at 24 hrs post infection. The mutant and wild type strains showed similar cell association and uptake as determined by confocal microscopy (data not shown). Complementation of the ΔABCH mutant with pAcpA and/or pHapA regained the majority of the ability to survive and replicate in all macrophages examined (<1 log reduction in CFU recovered at 24 hrs post-infection, data not shown).

We also investigated the survival of the Schu S4 acid phosphatase mutants in human neutrophils at 15 min to 120 min post infection (Fig. 2E). Survival in these cells showed a similar pattern to the survival observed in macrophages, with the ΔABCH mutant having a severe survival defect and the *ΔacpA* and *ΔhapA* showing intermediate survival defects (Fig. 2E). Additionally, these differences could also be observed at the earliest time point of 15 min in these cells (versus a similar time point in hMDMs) likely due to the increased antimicrobial action of human neutrophils vs. human macrophages.

### Mouse virulence analysis

**Figure 3:**
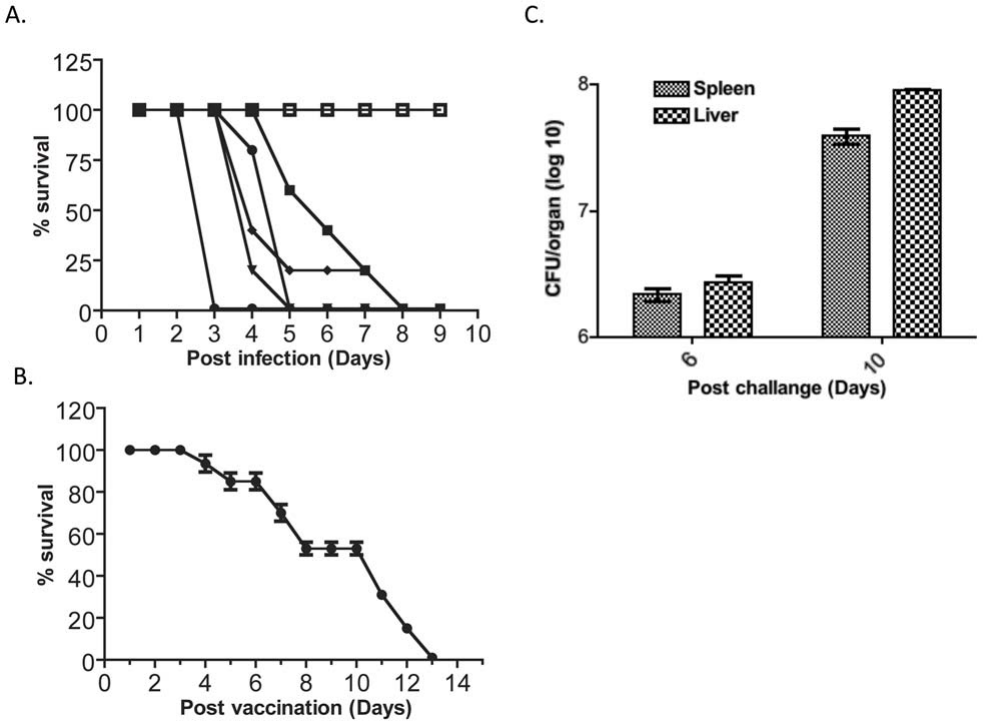
Mouse virulence assays. (A) BALB/c mice (N = 5) were anesthetized and infected with various *F. tularensis* Schu S4 wild type and the acid phosphatase mutants by the intranasal route (3×10^3^ CFU of *F. tularensis*). (▪), Δ*acpA* (▴), Δ*hapA* (▾), ACH (•), ΔABC (♦), ΔABCH (□). (B) Survival of ΔABCH vaccinated BALB/c mice (N = 17) challenged with *F. tularensis* Schu S4 wild type 42 days post vaccination. (C) Bacterial load in liver and spleen of BALB/c mice (N = 3) challenged intranasally with the *F. tularensis* Schu S4 wild type strain 42 days post vaccination with the ΔABCH strain (both at a dose of 10^3^ CFU). Mice were sacrificed at various time points post-*F. tularensis* Schu S4 infection to determine the fate of the challenge organisms.

To determine if any of the acid phosphatase mutants exhibit virulence defects in the mouse model of tularemia, 6–8 week old female BALB/c mice (n = 5) were infected with *ΔacpA*, *ΔacpB, ΔacpC*, *ΔhapA*, ΔAH, ΔCH, ΔABC, ΔACH, or ΔABCH (n = 20 for ΔABCH) and wild type Schu S4 strains by the intranasal route at a dose of ∼3×10^3^ CFU. Wild type, *ΔacpA*, *ΔacpB, ΔacpC*, *ΔhapA*, ΔAH, ΔCH and ΔABC infected mice died within 2–12 days after infection, with wild type mice succumbing first, followed by the single deletions and then the double/triple deletions (Fig. 3A). However, all mice infected with the ΔABCH mutant all survived for 6 weeks after infection. When a dose of 10^6^ CFU was used, mice of all strains tested died rapidly except those infected with the *Δ*ABCH strain, which showed 70% survival (data not shown). To further investigate the mouse virulence studies, we collected the liver and spleen of the infected mice and determined their bacterial load. As expected, a high bacterial burden (>10^7^ CFU) in the liver and spleen of the mouse was correlated with death in all tested strains (data not shown).

To determine whether the attenuated *Δ*ABCH strain was protective against Schu S4 wild type challenge, we infected all of the surviving *Δ*ABCH vaccinated mice (both 10^3^ and 10^6^ CFU) with Schu S4 at 42 days post-vaccination. All the mice succumbed to infection and died by day 13 post-challenge (Fig. 3B). At days 6 and 10 post-challenge, some mice were sacrificed and their organ burdens were determined. The results showed significant bacterial burdens in the liver and spleen of mice 6 days post challenge that increased over a log by 10 days post-challenge (Fig. 3C).

### TEM analysis of phagosomal escape

**Figure 4:**
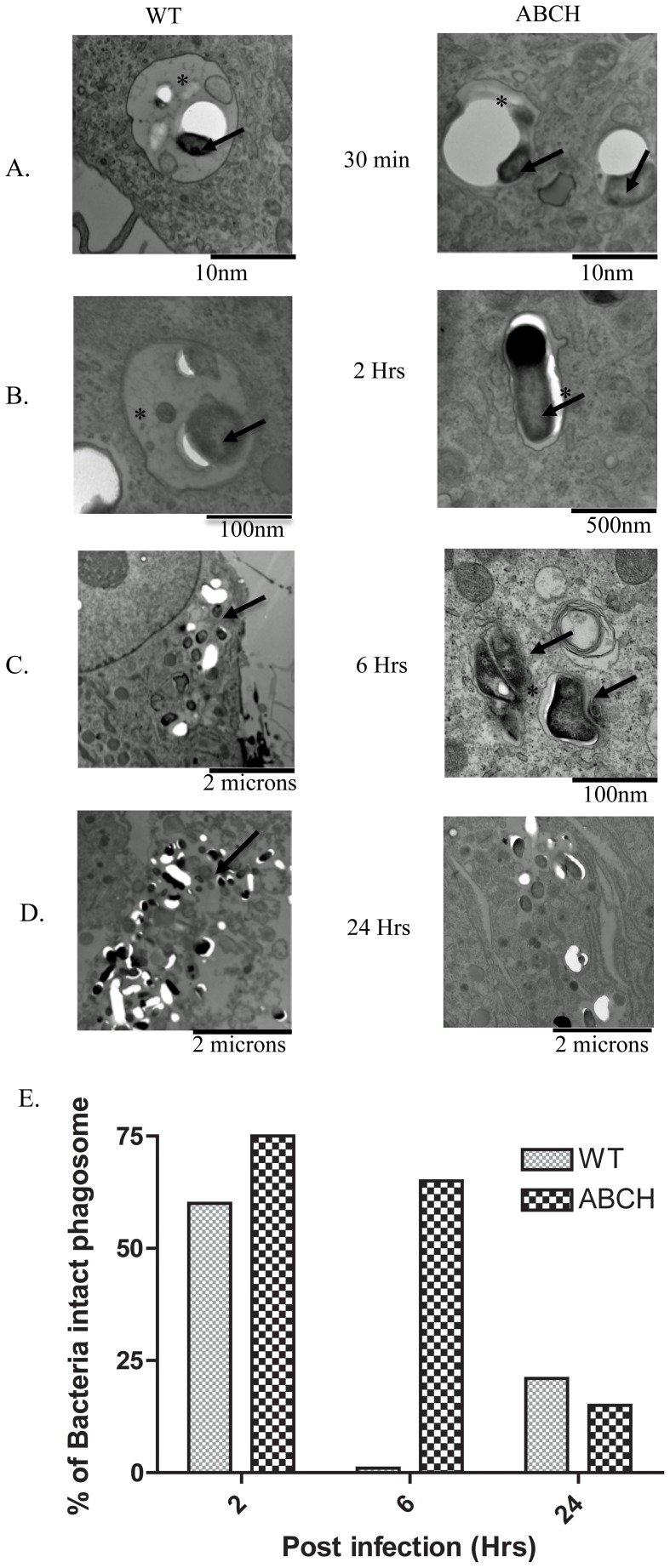
Transmission electron microscopy. Transmission electron microscopy images of PMA-induced THP-1 human macrophage-like cell lines infected with *F. tularensis* Schu S4 (left panel) and Δ*ABCH* (right panel) obtained at (A) 30 min, (B) 2 hours, (C) 6 hours, (D) 24 hours post-infection. (E) Quantitative assessment of bacteria within/outside of phagosomes in a minimum of 300 cross sections/test group (≥500 bacteria). An asterisk (*) represents the double membrane of a vacuole and the arrows point to representative bacteria in the macrophages.

To observe the uptake and trafficking of the Schu S4 wild type and *Δ*ABCH strains, we infected THP-1 macrophages and examined them by transmission electron microscopy at 30 min, 2, 6, 12, 18 and 24 hrs post infection. We found that at 30 min post infection, the bacteria that had been engulfed were uniformly enclosed in a membranous vesicle (Fig. 4A). By 2 hrs post infection, 60% of the wild type and 85% of *Δ*ABCH strains were still within phagosomes with an intact vacuolar membrane (Fig. 4B, E). However, at 6 and 12 hrs post infection essentially 100% of wild type bacteria were found in cytosol (Fig. 4C, E, data not shown). In contrast, 65- and 40% of the *Δ*ABCH bacteria were found in intact phagosome by 6 and 12 hr post infection, respectively (Fig. 4C, E, data not shown). By 18 hrs post infection 11% of wild type and 47% of *Δ*ABCH bacteria were within vacuoles with multilayer membranes (data not shown) and similar numbers at 24 hrs post infection (presumed autophagy vacuoles; Fig. 4D, E). These data demonstrate a partial phagosomal escape defect of the *F. tularensis* Schu S4 *Δ*ABCH mutant, likely resulting in the observed killing of this strain within macrophages,

### ROS production

**Figure 5:**
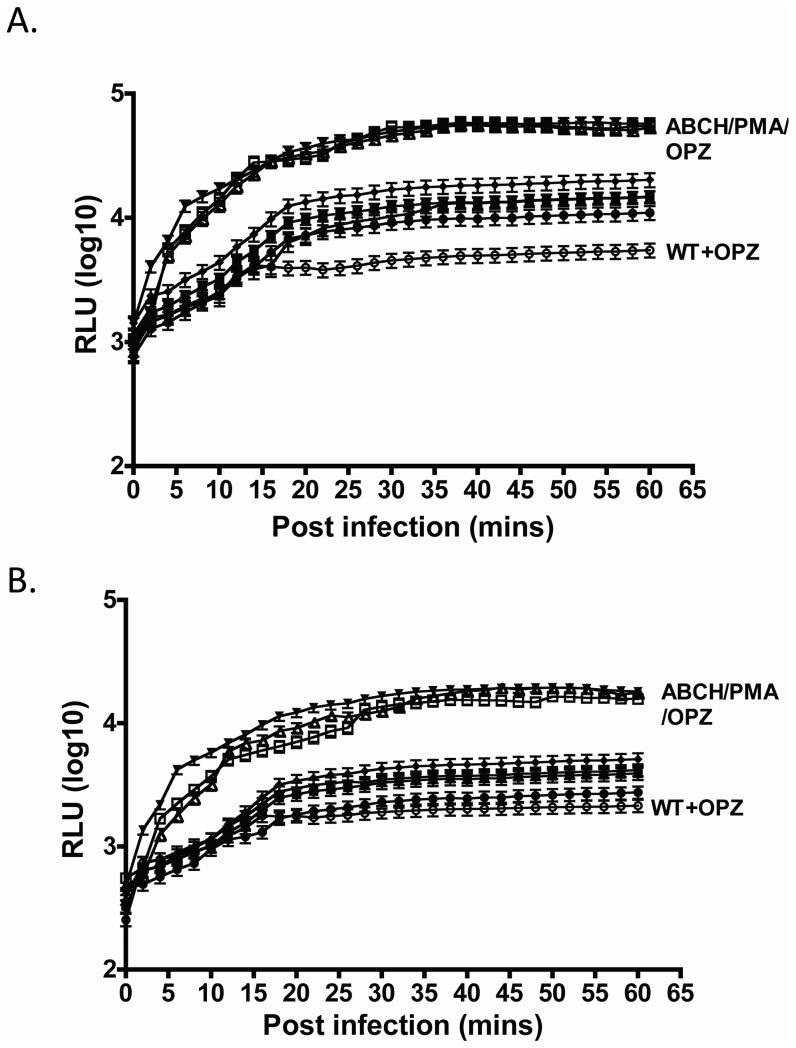
Detection of ROS production in human phagocytes. The luminescence was measured over a 60 min time period in human (A) neutrophils and (B) hMDMs, and the baseline ROS luminescence was determined by measuring the luminescence of control cells treated with medium only. *F. tularensis* Schu S4 (▪), Δ*acpA* (▴), Δ*hapA* (▾), formalin killed *F. tularensis* Schu S4 (⧫), *F. tularensis* Schu S4 and serum-opsonized zymosan beads (○), ΔABCH (□), PMA (Δ), serum-opsonized zymosan (▿).

Previous studies demonstrated that deletion of the acid phosphatases in *F. novicida* resulted in a loss of the ability of the wild type strain to suppress ROS production in human neutrophils and macrophages [49]. To determine whether *F. tularensis* Schu S4 acid phosphatase mutants also lack this suppressive ability in human phagocytes, we measured the production of ROS in infected human neutrophils and MDMs (60 min time course) using the luminescence probes luminol or lucigenin, respectively. As observed in our previous study and by others, *F. tularensis* Schu S4 induced minimal amount of ROS in neutrophils and hMDMs [66] (Fig. 5A and B). However, the formalin killed Schu S4, opsonized zymosan, and PMA stimulate robust ROS production in neutrophils and hMDMs (Fig. 5A and 5B). Phagocytes infected with the *ΔacpA* or *ΔhapA* mutant produced >12-fold increased ROS in hMDMs but resulted in no significant change in neutrophil ROS production after 60 min of infection. However, human neutrophils and hMDMs infected with ΔABCH strain induced a 5- and 56-fold increase in ROS compared to Schu S4 strain, respectively. The ΔABCH strain complemented with pAcpA and/or pHapA restored the ROS suppressive activity of the strain in both neutrophils and hMDMs. Thus, these data suggest that *Francisella* acid phosphatases, including the truncated HapA, contribute to suppression of ROS production in human phagocytes.

**Figure 6:**
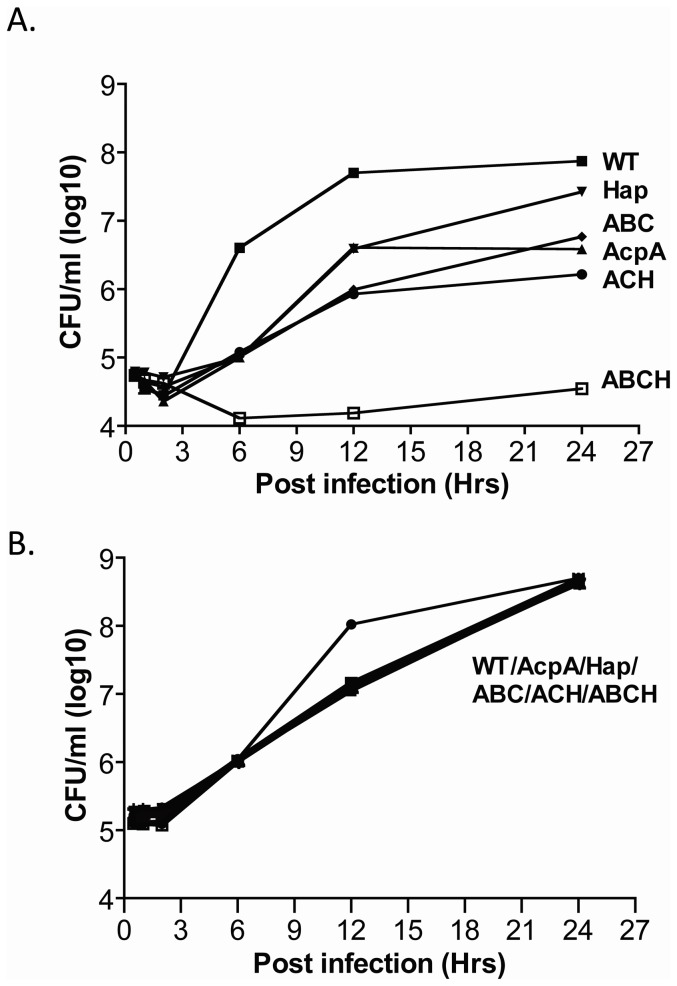
Intramacrophage survival of *F. tularensis* Schu S4 acid phosphatase mutants in BMM from p47*^phox+/+^* and p47*^phox-/-^* knockout mice. BMM were isolated from (A) p47*^phox+/+^* wild type C57BL/J mice and (B) p47*^phox-/-^* knockout mice C57BL/J infected with *F. tularensis* Schu S4 (▪) or acid phosphatase mutants Δ*acpA* (▴), Δ*hapA* (▾), ΔABC (♦), ΔACH (•), or ΔABCH (□) strains. Data are the mean ± SD of triplicate samples from one representative experiment (n = 2).

In our previous work, the *F. novicida* ΔABCH mutant was defective in survival within macrophages and neutrophils, and this was demonstrated to be the result of increased ROS production coupled with the dramatic loss in the ability to escape the phagosome. To examine the involvement of ROS in Schu S4 ΔABCH mutant phagocytic cell killing, survival was monitored in BMDM isolated from C57BL/6 and p47*^phox^* congenic knockout mice. The BMDMs isolated from wild type and the p47*^phox^* ^-/-^ mice were infected with Schu S4 wild type, *ΔacpA, ΔhapA,* ΔABC, ΔACH and ΔABCH strains, and the CFUs were compared at different time points. At 24 hrs post infection, the single and triple mutants examined showed intermediate survival defects in BMDMs of C57BL/6 wild type mice, while the ΔABCH mutant showed a dramatic survival defect (Fig. 6A). In contrast, all strains survived and replicated equally well in BMDM isolated from p47*^phox^* ^-/-^ mice (Fig. 6B). This suggests a strong correlation of intracellular survival defects in Schu S4 acid phosphatase mutants with NADPH oxidase-mediated ROS production and bacterial killing.

### Co-localization of NADPH oxidase components with *Francisella* in human neutrophils and macrophages

**Figure 7:**
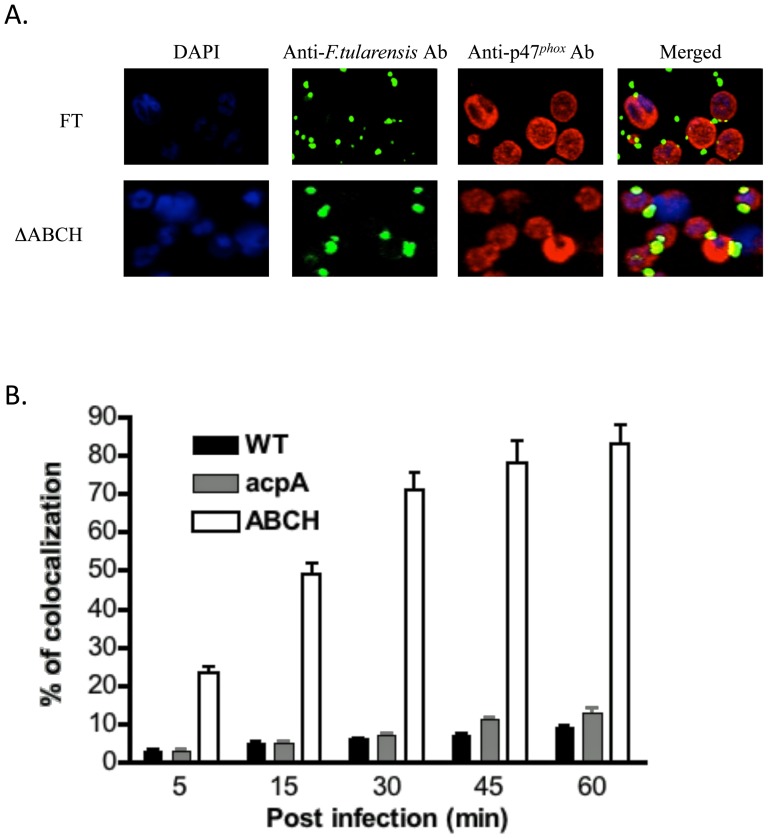
Co-localization of the *F. tularensis* Schu S4 wild type strain and mutant strains with p47*^phox^* in neutrophils. (A) Co-localization of the *F. tularensis* Schu S4 wild type and ΔABCH mutant strain with p47*^phox^* was determined at 5, 15, 30, 45 and 60 min post infection in neutrophils. *Francisellae* were detected following straining with goat anti-mouse Alexa Flour® 488 (green color) and p47*^phox^* was detected following staining with donkey anti-rabbit Alexa Fluor® 546 (red color). Representative confocal microscopy images of *F. tularensis* Schu S4 and ΔABCH co-localized with p47*^phox^* within neutrophils are shown at 30 min post infection. The images are representative of 1000 infected cells examined from triplicate cover slips in three independent experiments. (B) Co-localization of the *F. tularensis* Schu S4 wild type (black), Δ*acpA* (grey) and ΔABCH (white) mutant strains with p47*^phox^* was quantified at 5, 15, 30, 45 and 60 min post infection. Analyses were based on examination of 1000 infected cells examined from triplicate cover slips in three independent experiments. The results shown are cumulative data of five experiments (mean ± SD of triplicate samples in each test group).

**Figure 8:**
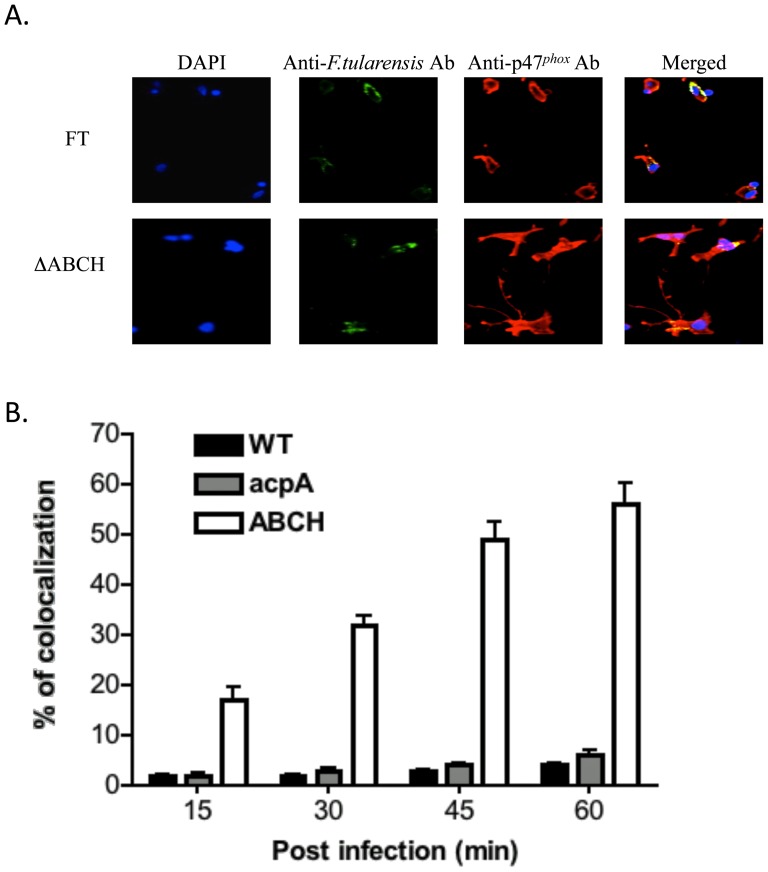
Co-localization of the *F. tularensis* Schu S4 wild type strain and mutant strains with p47*^phox^* in hMDMs. (A) Co-localization of the wild type and ΔABCH mutant strain with p47*^phox^* was determined at 5, 15, 30, 45 and 60 min post infection in neutrophils. *F. tularensis* Schu S4 strains were detected following straining with goat anti-mouse Alexa Flour® 488 (green color) and p47*^phox^* was detected following staining with donkey anti-rabbit Alexa Fluor® 546 (red color). Representative confocal microscopy images of *F. tularensis* Schu S4 and ΔABCH co-localized with p47*^phox^* within neutrophils are shown at 30 min post infection. The images are representative of 1000 infected cells examined from triplicate cover slips in three independent experiments. (B) Co-localization of the *F. tularensis* Schu S4 wild type (black), Δ*acpA* (grey) and ΔABCH (white) mutant strains with gp91*^phox^* was quantified at 5, 15, 30, 45 and 60 min post infection. Analyses were based on examination of 1000 infected cells examined from triplicate cover slips in three independent experiments. The results shown are cumulative data of five experiments (mean ± SD of triplicate samples in each test group).

Our previous work in *F. novicida* showed that acid phosphatase mutants co-localized extensively with NADPH oxidase components, as expected by their inability to suppress ROS production within professional phagocytes. Thus, we examined co-localization of Schu S4 wild type, Δ*acpA* and ΔABCH strains with p47*^phox^* of human neutrophils and MDMs by confocal microscopy. Representative confocal images of neutrophils with these strains are shown in Fig. 7A. Neutrophils infected with the ΔABCH strain showed an increasing co-localization with p47*^phox^* over time that reached its peak (83%) at 60 min post infection (Fig. 7B). The wild type Schu S4 and Δ*acpA* mutants co-localized poorly with p47*^phox^* (9 and 13%, respectively) at 60 min post infection. Similar, findings were observed in human MDMs infected with Schu S4, ΔABCH and *ΔacpA* mutants. Representative confocal images of infected human MDMs are shown at 60 min post-infection in Fig. 8A. The ΔABCH strain co-localized with p47*^phox^* with a maximum of 57% at 60 min post-infection, whereas the *ΔacpA* mutant and wild type Schu S4 showed a maximum of 6% and 4% co-localization at 60 min post infection, respectively (Fig. 8B). This suggests that phosphorylation of p47*^phox^* was suppressed by Schu S4 wild type strain but the loss of acid phosphatases (multiple but not single) is proposed to allow the p47*^phox^* components to phosphorylate and translocate to the membrane.

### Increased phosphorylation of NADPH oxidase components upon infection of neutrophils and hMDMs with the ΔABCH mutant strain

**Figure 9:**
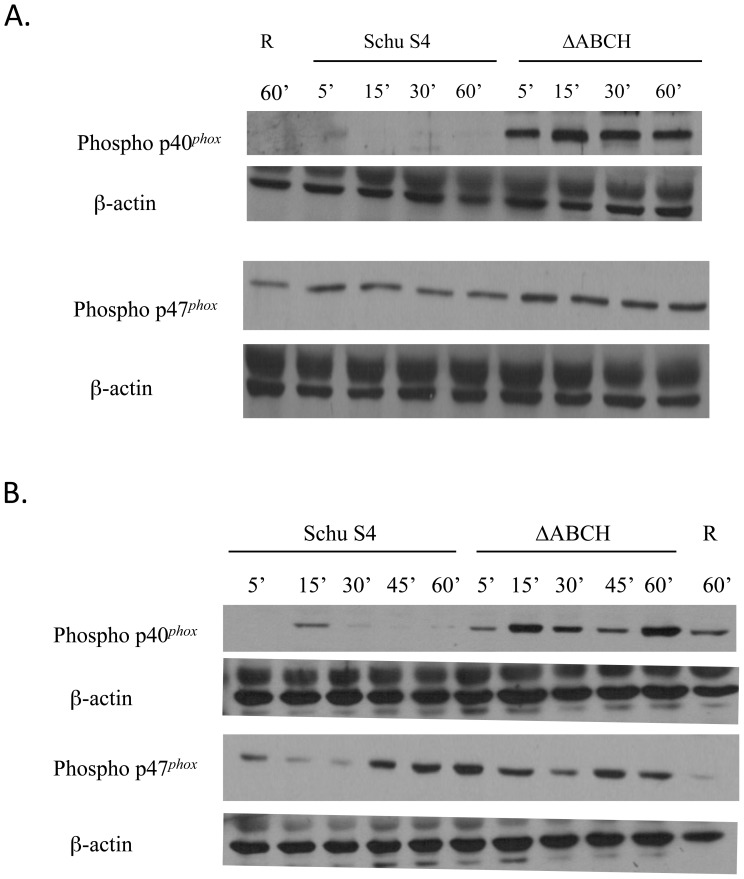
The effect of *F. tularensis* Schu S4 wild type and *acp* mutants on phosphorylation of p47*^phox^* and p40*^phox^* in human neutrophils and MDMs. (A) Neutrophils or (B) hMDMs were incubated with *F. tularensis* Schu S4 wild type or ΔABCH strains for the times shown. Lysates were loaded by protein equivalents, separated by SDS-PAGE and analyzed by Western blotting with antibodies specific for phosphorylated p47*^phox^* (rabbit anti-Pp47*^phox^*) and phosphorylated p40*^phox^* (rabbit anti-Pp40*^phox^*). The same membrane was re-probed with β-actin antibody to verify equal protein loading. Quantification of the ECL signal was measured by using a scanner and densitometry software. A representative Western blot image is shown (n = 7).

Previous data suggested that the *Francisella* acid phosphatases may directly or indirectly dephosphorylate NADPH oxidase components [49], [67], [68]. In addition, because phosphorylation of p47*^phox^* is required for its recruitment to the phagosome [69], [70], the above co-localization data suggests that the ΔABCH strain may lack the ability to dephosphorylate this and other NADPH oxidase components. To determine whether the enhanced ROS stimulation in neutrophils and hMDMs upon infection with ΔABCH mutant strain correlated with increased phosphorylation of NADPH oxidase complex subunits (likely caused by a lack of de-phosphorylation), we examined the phosphorylation of p47*^phox^* and p40*^phox^* during the course of infection. Neutrophils or hMDMs were infected with Schu S4 and ΔABCH mutant strains, cells were lysed at different time intervals, and the phosphorylation of p47*^phox^* or p40*^phox^* was detected by Western blot, using phospho-p47*^phox^* and phospho-p40*^phox^* antibodies. Neutrophils infected with the ΔABCH strain resulted in a rapid increase in phosphorylation of p47*^phox^* and p40*^phox^* within 5 min of infection compared to the Schu S4 strain (Fig. 9A). Similarly, hMDMs infected with the ΔABCH strain showed an increased phosphorylation of p47*^phox^* and p40*^phox^* by 15 min of infection (Fig. 9B). These results suggest that in Schu S4, the NADPH oxidase components are either phosphorylated more effectively or are no longer dephosphorylated in the absence of the *Francisella* acid phosphatases.

## Discussion

Acid phosphatases are ubiquitous in nature and hydrolyze the phosphoryl groups of phosphomonoesters at an acidic pH [71]. These enzymes are essential for mobilization of inorganic phosphates and in phospho-relay systems involved in signal transduction pathways in both prokaryotes and eukaryotes. Acid phosphatase enzymes from various pathogens (*Leishmania* spp, *Legionella* spp, *Bordetella* spp and *Francisella* spp.) are known or suspected to be involved in the virulence by inhibiting reactive oxygen species production in host cells [54]–[56], [58], [72].

Our previous study has shown that deletion of four acid phosphatases (ΔABCH) in *F. novicida* play a major role in phagosomal escape, intramacrophage survival, virulence in mouse model, induction of ROS production in human phagocytes and co-localization with the NADPH oxidase components of neutrophils and macrophages [23], [49]. A study performed by Child et al. illustrated that deletion of AcpA, AcpB and AcpC (ΔABC) in *F. tularensis* SchuS4 did not contribute to virulence in mouse and human macrophages [50]. Similar findings were observed in the ΔABC *F. novicida* mutant generated by Mohapatra et al. but the additional deletion of HapA in *F. novicida* (ΔABCH) resulted in attenuation of virulence [23]. The Child et al. study did not delete HapA in Schu S4 because it contains a C-terminal disruption by an insertion element, which would eliminate the predicted active site of the produced protein [50]. However, a recent study revealed that the truncated purified HapA protein from *F. tularensis* Schu S4 is functional and highly active at low pH in the presence of magnesium, iron and cobalt [64]. In the present study, we constructed deletion mutants of AcpA, AcpB, AcpC and HapA in the *F. tularensis* Schu S4 strain to determine if these acid phosphatases contributed to virulence.

In this study, we successfully deleted four acid phosphatases (*acpA, acpB, acpC* and truncated *hapA)* in *F. tularensis* Schu S4 strain. This mutant did not show any defect in growth in MMH broth or abnormal colony morphology on plates. It is clear from acid phosphatase activities measured in the various single, double, triple and quadruple mutant strains that all of the acid phosphatases, including HapA, contributed to the acid phosphatase activity of *F. tularensis* Schu S4. Though several combinations of *acp* deletions eliminate most of the acid phosphatase activity of *F. tularensis* Schu S4, this does not carry over to complete loss of other phenotypes such as intracellular replication and virulence. The *in vivo* phenotypes should be viewed independently as they involve documented factors unrelated to *in vitro* bacterial acid phosphatase activity such as Acp secretion/translocation and *in vivo* gene induction.

*Francisella* primarily targets host macrophages for their survival and replication. To examine the affect on virulence of all acid phosphatase mutants, we infected human and mouse macrophages (primary and cell lines) with acid phosphatase mutants. The *ΔhapA* strain was defective for survival in various macrophages by >10-fold compared to the wild type strain. Similarly, *Δ*ACH, *Δ*ABC and *Δ*ABCH mutants showed >20-1200-fold decrease in CFU numbers at 24 hours post-infection compared to wild type strain. The complementation of *hapA* and/or *acpA* in the *Δ*ABCH mutant partially restored the virulence, clearly indicating that at least these two acid phosphatases of *F. tularensis* Schu S4 are active, functional and contribute to *Francisella* virulence phenotypes.

To further explore the survival and growth of these acid phosphatase mutants *in vivo*, mice were infected intranasally. We found that the mice infected with single, double or triple acid phosphatase mutants survived longer than wild type strain but died by day 5–8. This result was similar to our previous result with *F. novicida* and that of Child et al. with *F. tularensis* Schu S4 [50]. However, mice infected with *Δ*ABCH (3×10^3^ CFU; 2-3-logs above the LD_50_) were 100% attenuated, and those infected with *Δ*ABCH at a dose of 10^6^ CFU resulted in 70% mouse survival at 42 days post-infection. When these vaccinated mice were challenged with the wild type strain, all succumbed by 13 days post-infection. It is unclear why the *Δ*ABCH strain of *F. novicida* was 100% protective upon challenge while the *F. tularensis* Schu S4 mutant was not. However, the above data strongly support that the additional deletion of the truncated *hapA* in the *Δ*ABC strain contributes to *F. tularensis* Schu S4 pathogenesis *in vitro* and *in vivo*.

Several studies have been shown that *Francisella* spp. induce the formation of spacious pseudopod loops for phagocytosis followed by a sequential phagosomal maturation process and escape of *Francisella* into host cytosol within 2–6 hours post-infection [15], [73]. There are likely several virulence factors involved in *Francisella* phagosomal escape from the *Francisella* containing vacuole, but the mechanism behind this process has yet to be resolved [19], [23], [74]–[77]. To better understand the mechanism and view the niche of the *Δ*ABCH strain in host phagocytes, we performed transmission electron microscopy of THP-1 macrophages infected with wild type and acid phosphatase mutants. Our data demonstrated that the wild type strain escaped the phagosome and replicated in the host cytosol by 6 hours post-infection, whereas ∼50% of the *Δ*ABCH mutant were residing in a an intact vacuole at 24 hour post infection. These data suggest that the *Francisella* acid phosphatases contribute to the kinetics of phagosomal membrane disruption, and the inability of the *Δ*ABCH strain to escape from the phagosome and replicate in the cytosol. It is noted that the *F. novicida Δ*ABCH mutant had a much greater defect in phagosomal escape, which could be due to the activation or involvement of novel factors present and potentially secreted during *F. tularensis* Schu S4 phagosomal residence.

Our previous studies demonstrated that the collective deletion of four acid phosphatases in *F. novicida*, unlike the wild type strain, induced the oxidative burst in human neutrophils and monocyte derived macrophages [49]. In *F. tularensis* Schu S4, deletion of these four acid phosphatases also resulted in significant negative effects on virulence-related phenotypes. Furthermore, AcpA is secreted from *F. tularensis* SchuS4 *in vitro* and *in vivo* and purified acid phosphatases from *Francisella* dephosphorylate the NADPH oxidase components p40^phox^ and p47^phox^ [49], [62]. To determine if the *F. tularensis* Schu S4 acid phosphatases affect the oxidative burst and to further elucidate the mechanisms behind the attenuation and survival of the *F. tularensis* Schu S4 *Δ*ABCH mutant *in vivo* and *in vitro*, we examined ROS induction in infected human neutrophils and MDMs. Our data illustrated that the *Δ*ABCH strain induced a robust oxidative burst and increased ROS production in human MDMs and neutrophils by 5- and 56-fold, respectively versus the wild type strain. Additionally we observed that more than 50% of the *Δ*ABCH strain co-localized with the p47^phox^ NADPH component in human neutrophils and MDMs at a time point in which <5% of the wild type strain similarly co-localized. These data suggest that *F. tularensis* Schu S4 acid phosphatases are involved in ROS suppression in human phagocytes.

To further understand the necessity and the link between *Francisella* acid phosphatases with NADPH oxidase components, we isolated the BMDMs from p47^phox-/-^ and wild type mice and infected them with the *F. tularensis* Schu S4 and *Δ*ABCH strains. This revealed that the *Δ*ABCH strain was severely defective in survival within BMDMs isolated from wild type mice; however, there was no survival defect observed in BMDMs isolated from in p47^phox-/-^ at 24 hours post infection. Additionally, Western blot analysis showed that the neutrophils and hMDMs infected with the *Δ*ABCH strain resulted in a marked increase in phosphorylation of p40^phox^/p47^phox^ as early as 5 minutes post-infection. Our previous study and the present data suggest that, regarding the *Δ*ABCH strain, the relative increase in p47^phox^ and p40^phox^ phosphorylation and association with NADPH oxidase components inside the host phagocytes is a result of the inability to mount an acid phosphatase-mediated dephosphorylation of NADPH oxidase components. This results in an increased ROS response and decreased bacterial survival in phagocytes.
