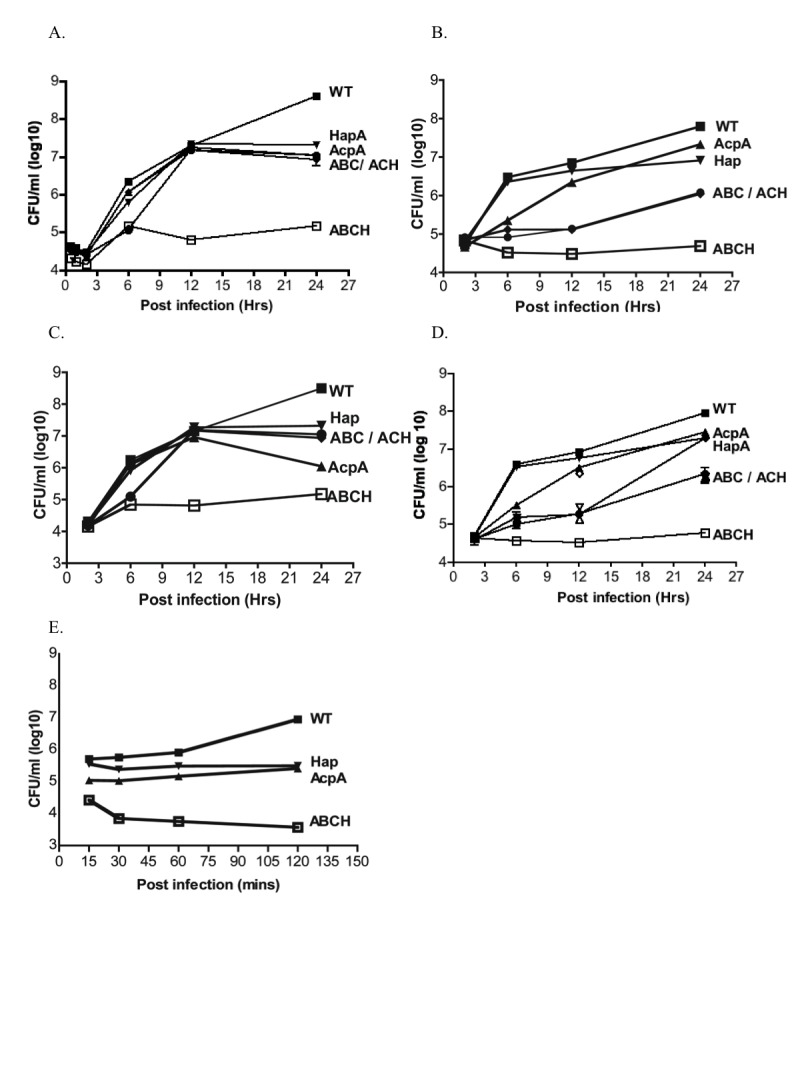# Correction: Type A *Francisella tularensis* Acid Phosphatases Contribute to Pathogenesis

**DOI:** 10.1371/annotation/67f7aa15-31d8-4760-9dfc-4f4e0bd63ea9

**Published:** 2014-01-13

**Authors:** Nrusingh P. Mohapatra, Shilpa Soni, Murugesan V. S. Rajaram, Kristi L. Strandberg, John S. Gunn

The is an error in the panels for Figure 2. Please view the correct Figure 2 here: 

**Figure:**